# Prevalence of Overweight, Obesity, and Abdominal Obesity in Polish Adults: Sociodemographic Analysis from the 2016–2020 National Health Program

**DOI:** 10.3390/nu16234248

**Published:** 2024-12-09

**Authors:** Iwona Traczyk, Alicja Kucharska, Beata I. Sińska, Mariusz Panczyk, Piotr Samel-Kowalik, Anna Kłak, Filip Raciborski, Mariusz Wyleżoł, Bolesław Samoliński, Dorota Szostak-Węgierek

**Affiliations:** 1 Department of Public Health, Faculty of Health Sciences, Medical University of Warsaw, 02-091 Warsaw, Poland; iwona.traczyk@wum.edu.pl; 2 Department of Human Nutrition, Faculty of Health Sciences, Medical University of Warsaw, 02-091 Warsaw, Poland; alicja.kucharska@wum.edu.pl (A.K.); beata.sinska@wum.edu.pl (B.I.S.); 3 Department of Education and Research in Health Sciences, Medical University of Warsaw, 02-091 Warsaw, Poland; mariusz.panczyk@wum.edu.pl; 4 Department of the Prevention of Environmental Hazards, Allergology and Immunology, Faculty of Health Sciences, Medical University of Warsaw, 02-091 Warsaw, Poland; piotr.samel-kowalik@wum.edu.pl (P.S.-K.); filip.raciborski@wum.edu.pl (F.R.); boleslaw.samolinski@wum.edu.pl (B.S.); 5 Department of General, Vascular and Oncological Surgery, Medical University of Warsaw, 02-091 Warsaw, Poland; wylezolm@hotmail.com; 6 Department of Clinical Dietetics, Faculty of Health Sciences, Medical University of Warsaw, 02-091 Warsaw, Poland; dorota.szostak-wegierek@wum.edu.pl

**Keywords:** adult Polish population, anthropometric measurements, obesity, overweight, prevalence

## Abstract

Introduction: Excessive body weight, particularly the abdominal distribution of adipose tissue, has remained an important public health concern for years. Objectives: The study aimed to assess the prevalence of general overweight status and obesity, as well as abdominal overweight status and obesity in the adult Polish population. Material and methods: The results are based on the analysis of anthropometric data of 3735 people aged 19 and over who were surveyed under the 2016–2020 National Health Program. Results: Excessive body weight (BMI > 25 kg/m^2^) was reported in 56.6% of the respondents. Abdominal overweight was found in 20.8% of respondents and abdominal obesity in 31.7% of the respondents. Abdominal obesity was more common in women than in men (44.3% vs. 18.0%, *p* < 0.001). Over half of women over 55 (approx. 60%) had abdominal obesity. The odds of becoming overweight/obese as measured by the BMI depended on the age and sex of the respondents. It was confirmed that men were over 28% more likely to develop such a condition than women (OR = 1.288, *p* < 0.001). The odds increased with age (OR = 1.029, *p* < 0.001). The type of person with obesity/found to be overweight in Polish population was most often a rural resident, aged 65.0–74.9, assessing their financial situation as average, being married/in a partnership, and not declaring the occurrence of cardiovascular diseases. The person with an overweight status had secondary education, and the person with obesity had primary/lower secondary/vocational education. Conclusions: Being overweight and having obesity, both general and abdominal, are still a major epidemiological problem in Poland. The results obtained in this study suggest that the prevalence of being overweight and having obesity has decreased over the last 10 years, though this assumption requires further investigation. Rural residents with lower education should be covered by educational activities adapted to their needs and possibilities, considering that obesity in young women, especially when having abdominal obesity, may be associated with an increased risk of infertility caused by ovulation disorders. Further research and educational activities are necessary. Excessive body weight affected almost 42% of the women from the youngest age group, and abdominal obesity was found in 21% of them.

## 1. Introduction

Excessive body weight, especially obesity, exerts an enormous negative effect on the lives of people affected by the pathology, both in the social and health aspects. Obesity is a severe metabolic disease that may lead to disability and premature death by increasing the risk of developing numerous other serious diseases, including cardiovascular diseases, type 2 diabetes, hypertension, dyslipidemia, and some cancers [1,2,3,4]. Obesity is associated with a host of physical health issues [5]. Moreover, individuals living with obesity often face social exclusion and loneliness, which may contribute to mental health issues such as depression, although the two can co-occur [6]. The discussion about the excessive spread of being overweight and being obese in the world has been going on for many years [7]. According to the data published so far, the prevalence of being overweight and being obese has also increased in Poland. At the end of the 20th century, excessive body weight affected more than half of the adult population of Poland (men at 56.7% vs. women at 48.6%) [8]. In 2005, the values increased and amounted to 60.2% in men and 50% in women. A decade later, an increase in the number of overweight and people with obesity was noted again, i.e., in 2013–2014, 67.3% of men and 52.9% of women were affected, including obesity measured in 24.4% of men and 25.0% of women [9].

In the second decade of the twenty-first century, two large educational projects were carried out in Poland, the aim of which was to prevent becoming overweight and the spread of obesity in the adult Polish population: (Pol-Health) [10] and Keep the Balance [11]. It is difficult to determine to what extent these projects and many other smaller ones implemented in Poland have had in terms of impact on the current frequency of the discussed phenomenon in Poland. However, our research, being a part of the 2016–2019 NPZ project, indicates that the prevalence of being overweight and having obesity in the 19–64 age group is currently 51%. Although we confirmed that this phenomenon is still more common in the group of men than women (55 vs. 47%), it may suggest a decrease in the overall prevalence of excess body weight among adult residents of Poland [12]. The results of these recent studies prompted us to conduct an in-depth analysis of anthropometric data in the population of Polish adults aged 19 and over. The study aimed to assess the prevalence of general overweight and obesity diagnoses, as well as abdominal overweight and obesity diagnoses in the adult Polish population.

## 2. Material and Methods

### 2.1. Study Design and Patients Selection

The data analyzed in the present study were obtained from two representative cross-sectional studies of the dietary habits and nutritional status reports of the adult Polish population conducted in the years 2017–2020 within the National Health Program (NPZ 01 and NPZ 02) financed by the Ministry of Health. The NPZ 02 program included 2000 people aged 19–65, and the NPZ program 01 included 1735 people aged over 65. All analyses of anthropometric data concerned 3735 people. Age-standardized prevalence rates were calculated with the direct method using weights assessed for the population of Poland based on the data of Statistics Poland as of 31 December 2022 [13]. The weighted size of the study group was 2061, including 985 men aged 19+. Prior to the COVID-19 pandemic (October 2017–March 2020), all data were collected using the Computer-Assisted Personal Interviews (CAPIs) method. In the period from June to December 2020, the telephone interview method (CATI) was used. The number of respondents who completed the study prior to the COVID-19 pandemic was 90% of individuals.

### 2.2. Data Collection and Instruments

Anthropometric indicators were calculated on the basis of data obtained from measurements taken by interviewers, i.e., body weight, height, and waist circumference (WC). The measurements were performed according to established standards. Portable Omron electronic scales (HBF-212 model) were used to measure body weight (with an accuracy of 0.1 kg). Body height measurements (with an accuracy of 0.1 cm) were performed using a graduated anthropometric tape for linear tests and a set square. The same anthropometric tape was used to measure waist circumference. General overweight definition was defined as a BMI value from 25 to 29.9 kg/m^2^, and general obesity as a BMI of 30 kg/m^2^ or higher. In addition, for respondents aged 65 and older, the Lipschitz criteria were used, according to which excessive body weight in seniors is diagnosed with the BMI of >27 kg/m^2^ [14]. According to European guidelines, waist circumference of 94 to 101.9 cm in men and of 80 to 87.9 cm in women was classified as abdominal overweight and that of 102 cm or higher in men and 88 cm or higher in women as abdominal obesity [15]. The crude prevalence rate of general and abdominal obesity and overweight status with 95% confidence interval (CI) was calculated for the following age groups: 19–34.9, 35–44.9, 45.0–54.9, 55–64.9, 65.0–74.9, and 75 years and older in men and women.

During the pandemic, when direct contact with the respondents was impossible, the necessary anthropometric data were obtained based on the respondents’ declarations. A detailed description of the anthropometric measurements performed is provided in another publication [16].

### 2.3. Statistical Analysis

In this study, BMI and WC indices were analyzed using comprehensive descriptive statistical methods. The BMI data were articulated in terms of the mean, accompanied by a 95% confidence interval (CI) and standard deviation (SD), with stratification across sex and age groups. Additionally, specific percentile values were calculated. Frequency distributions for the BMI and WC categories were reported in numbers (*n*) and percentages (%), employing bootstrapping (10.000 iterations) for determining the 95% CI for each frequency.

The application of weights that corresponded to the actual frequencies of characteristics such as the sex, age, place of residence, and educational level in the Polish population was a crucial aspect of the analysis. These weights were adapted based on the data from the latest 2021 National Population and Housing Census conducted by the Statistics Poland [13], ensuring the representativeness of the sample.

For inferential statistical analysis, a null hypothesis testing approach was adopted. The comparison of mean BMI values across different groups was conducted using the Analysis of Variance (ANOVA) with the Games–Howell post hoc test. The Mann–Whitney U test with tie correction was utilized to examine differences in the frequency across various BMI or WC categories, with the eta-squared (η^2^) measure providing the effect size.

Logistic regression modeling was applied to estimate the probability of developing excess weight or obesity, including abdominal obesity. The model parameters were estimated using the maximum likelihood method, providing ORs and 95% CIs for sex and age as independent variables. Furthermore, an adjusted OR was calculated in a multivariate regression model, considering additional variables like the place of residence, education, financial status, marital status, professional situation, and the presence of cardiovascular diseases. Predictors were selected for inclusion in the model based on their clinical relevance and prior evidence of association with being overweight and having obesity from the literature. All selected variables were entered into the logistic regression model simultaneously using the enter method (forced entry). To ensure the model’s validity, we assessed multicollinearity among the independent variables using Variance Inflation Factors (VIFs). All VIF values were below 5, indicating no significant multicollinearity issues. The goodness-of-fit of the model was evaluated using the Akaike Information Criterion (AIC). Detailed diagnostic results are provided in the Supplementary Materials.

Data completeness was ensured in this study, as the target sample size (*n* = 3735) was achieved with complete responses to all questionnaire items from all participants. Therefore, there were no missing or erroneous data to handle in the analysis.

All statistical analyses were carried out using STATISTICA™ version 13.3 (TIBCO^®^ Software Inc., Palo Alto, CA, USA). A 5% level of significance was set for rejecting the null hypothesis in all statistical tests.

### 2.4. Ethical Considerations

This study was approved by the Ethical Review Board at the Medical University of Warsaw (approval No. AKBE/163/17 and AKBE/164/17). The study was conducted in accordance with the General Data Protection Regulation [17].

## 3. Results

Sociodemographic sample characteristics by sex in Poland (2017–2020) are presented in Table 1. The respondents differed significantly in terms of sex depending on their place of residence, level of education, current financial situation of the family, marital status, selected professional situations, and the occurrence of cardiovascular diseases.

**Figure 1:**
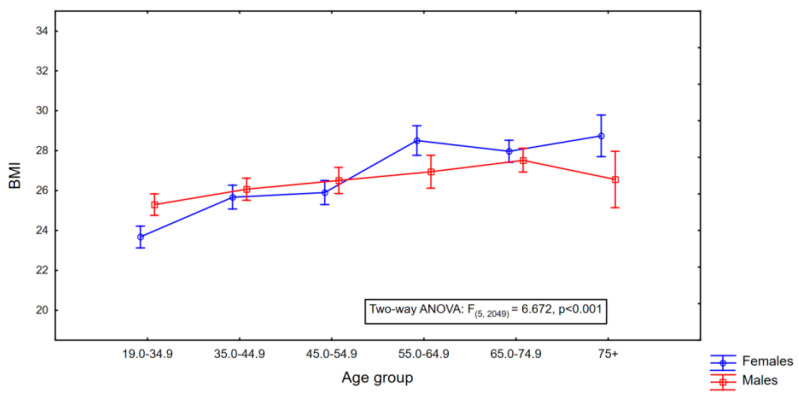
Sex- and age-related differences in mean body mass index (BMI) among a sample of Polish adults. The midpoints represent the mean BMI values for each age group, and the vertical lines indicate the 95% confidence intervals.

There were no statistically significant differences between the mean BMI values in the group of men and women (M: 26.40 (±5.17) vs. F: 26.26 (±3.71), t = 0.804, *p* = 0.421) (Table 2). An increase in the average BMI value was observed in both the men and women groups (Table 3), but in the women’s group, it was stronger ((F: 5.664), t = 7.110, *p* < 0.001) (Figure 1).

The healthy weight range was recorded in 41.8% of the study population. The overweight and obesity (BMI > 25 kg/m^2^) ranges were found in 56.6% of respondents, while 1.63% had too-low body weight. In the group of men overall, a higher percentage of people with excessive body weight was recorded than in the group of women (60.0 vs. 53.7%).

The analysis of the prevalence of being overweight and being obese measured by BMI categories in age groups showed that the prevalence of excess body weight increased with the age of the respondents until the age of 64.9, when it reached the value of 73.1% and decreased slightly in senior age. In all age groups, men were more likely than women to be overweight. However, women with obesity were significantly more likely than men (except for people aged 35.0–44.9 years) when the difference did not show statistical significance (Table 4). Analyzing the prevalence of excess body weight in seniors based on the Lipschitz criteria, the percentage of people with BMI > 27 kg/m^2^ exceeded 50%, except for the oldest men, in whom excess body weight occurred less frequently and affected 44.4% of people.

**Figure 2:**
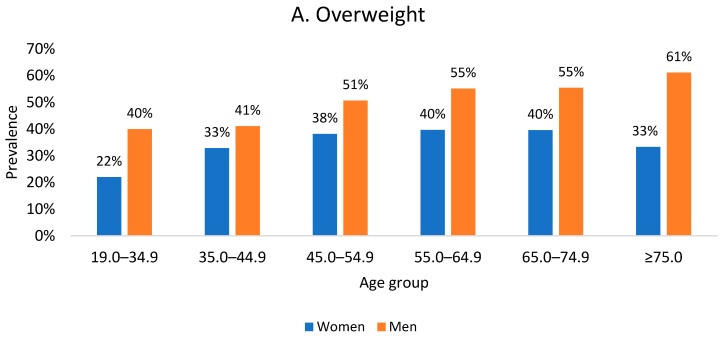

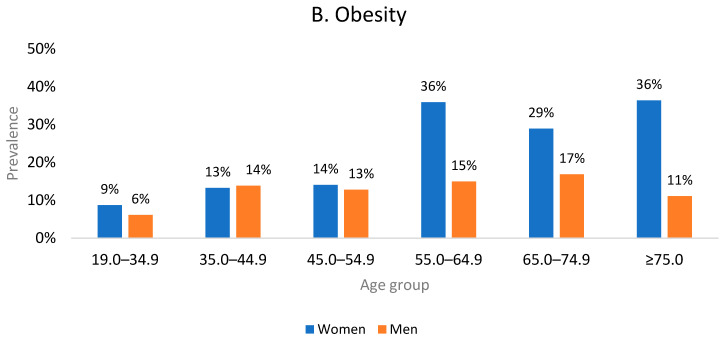
Prevalence of being overweight (**A**) and being obese (**B**) in the study group depending on age and gender.

Figure 2 presents the prevalence of being overweight and being obese in age groups. The data clearly show how the prevalence of being overweight and being obese in Poland changes with age depending on gender. The percentage of women with excessive body weight in the youngest analyzed age group was just over 30%; at the same age, the percentage of men with excessive body weight reached 46.1%, of which obesity occurred in 8.7% of women and 6.1% of men, respectively. In respondents 10 years and older (aged 35–44.9), the prevalence of obesity reached nearly 14% in men and 13.2% in women.

The percentage of adults with obesity in Poland up to 55 years of age in the analyzed age groups was at a maximum of 14.1% in the group of women aged 45–54.9 years and 13.9% in the group of men aged 35–44.9 years. In the following decades of life, in the group of men, the prevalence of obesity increased to 16.8% (age 65.0–74.9 years) and decreased to 11.1% in the oldest seniors. Women aged 55 and older struggled with obesity much more often than men; this was especially true for women aged 55–64.9 and seniors aged ≥75, where obesity exceeded 36%. Th overweight status, in turn, dominated in the group of men. The highest percentage of overweight men (61.1%) was recorded in the group of the oldest seniors. In the group of women, the most overweight people (39.7%) were aged 55.0–64.9.

The analysis of the prevalence of abdominal overweight and abdominal obesity measured by waist circumference showed that they concerned 20.8% and 31.7% of the respondents, respectively. In the group of women, a significantly higher percentage of individuals with abdominal obesity was noted compared to the group of men (44.3% vs. 18.0%, *p* < 0.001). The percentages of women with abdominal overweight were similar across all age groups and ranged from 18.3% to 24.2%. An exception was noted in the group of women aged 45–54.9 years, in whom the prevalence of abdominal overweight cases was 29.7%. In the case of men, the lowest percentage of individuals with abdominal overweight was found in the group over 75 years of age (13.9%), and the highest was found among men aged 55–64.9 years (24.3%).

The highest percentage of people with abdominal obesity was found in women over 55 years of age (approx. 60%). In this age group, abdominal obesity was significantly less common among men (20–30%) (Table 5). 

### Chances of Developing Overweight or Obesity

Analysis with the logistic regression model indicated that the odds of developing excess weight/obesity measured by the BMI index were dependent on the age and sex of the respondents. It was confirmed that men were over 28% more likely to develop such a condition than women (OR = 1.288, *p* < 0.001). In addition, the odds also increased with age (OR = 1.029, *p* < 0.001) (Table 6).

The analysis of the prevalence of being overweight comprising sociodemographic variables showed that an overweight person was most often a rural resident, aged 65.0–74.9, with secondary education, assessing their financial situation as average, remaining in a marriage/partnership, and not declaring the occurrence of cardiovascular diseases (Table 7). The analysis of obesity prevalence comprising sociodemographic variables revealed that a person with obesity was most often a person living in rural areas, aged 65.0–74.9, with primary/lower secondary/vocational education, assessing their financial situation as average, being married/in a partnership, and not declaring the occurrence of cardiovascular diseases (Table 7).

## 4. Discussion

Overweight conditions and obesity still remain a serious problem in Poland. Our research showed that excessive body weight affected 56.8% of people aged 19 and over, including obesity in 16.4%. Excessive body weight was more prevalent in men than in women (60.2 vs. 53.1%), while obesity was much more common in women (20% vs. 12.4%). The percentage of individuals with excessive body weight exceeded the average for the European Union countries, which was 52.7% in 2019 [18] and lower than the value (58%) provided in the quoted Eurostat report. The neighboring countries also struggle with a high prevalence of overweight diagnoses and obesity. In the Czech Republic, this phenomenon affects 60% of the population: 59% in Slovakia, 54% in Germany, and 57% in Lithuania. The percentage of people who are overweight and people suffering from obesity was below 50% in Italy (46%) and France (47%). The analysis of research conducted worldwide indicates that being overweight and being obese still remain an important public health problem in the United States (70%) [19], Australia (67%) [20], and Canada (65%) [21]. Chinese research published in 2023 indicated that almost half of adult residents (48.9%) were characterized by excessive body weight [22]. Despite the high prevalence of overweight cases and obesity cases in the contemporary world, this does not mean that it cannot be otherwise. This was highlighted by Japanese data, according to which excessive body weight occurred in 24% of the population, including obesity in 4% [23].

In Poland, in the years 2003–2014, the indices of excessive body weight increased. In 2003–2005, obesity was noted in 20.8% of men and 22.4% of women [24], and several years later (2013–2014), it was reported in 23.2% of men and 23.1% of women [9]. Excessive body weight in 2013–2014 occurred in 67.6% of men and 55.5% of women. Seemingly, the lower increase in the prevalence of overweight cases and obesity nowadays may be due to the growing interest in a healthy lifestyle, being a result of the educational programs mentioned earlier, as well as the improvement of the financial situation of the inhabitants of Poland [25]. However, it is too early to talk about a downward trend, although even stopping the increase in this phenomenon should be considered beneficial. As regards further preventive measures, attention should be paid to the problem of the increase in the frequency of excessive body weight with age. The lowest percentage of overweight and people with obesity (38.5%) occurred in the group of respondents aged 19–34.9 years, and the highest percentage (73.1%) in those aged 55–64.9 years. It decreased only slightly until late old age (70.6% of people had a BMI ≥ 25 kg/m^2^). Data on the body mass index in the oldest respondents were also analyzed in relation to the Lipschitz classification [14], according to which BMI ranging from 22.0 to 27.0 kg/m^2^ in senior age is considered normal. Values above 27.0 kg/m^2^ indicate excessive body weight. In the study group, 51.3% of people aged 65.0–74.9 and 51.0% of people aged ≥ 75 years met the criteria of excessive body weight according to the Lipschitz classification. Research showed that the low body weight of seniors was related to the occurrence of frailty syndrome, and a high BMI would be unfavorable due to the increased risk of functional disability and coexisting factors of weakness [26]. The comparison of the frequency of underweight cases in seniors according to the WHO and Lipschitz criteria indicates slight differences, i.e., in people aged 65–74.9 years, it was 0.7% and 6.2%, and in people ≥ 75 years, it was 0.0% and 2.9%, respectively, which clearly indicates that excessive body weight is a more common problem among Polish seniors. However, it does not exclude the problem of frailty syndrome, because a significant percentage of older people have sarcopenic obesity. Depending on the diagnostic criteria, the prevalence, of sarcopenia in people over 60 years of age was 10–27% [27], and for sarcopenic obesity, it was 11% [28].

Research showed that the prevalence of excessive body weight varied according to sex. In Poland, being overweight is more common in men, while obesity dominates in women, and the differences increase with age. In the youngest age group, obesity affected 8.7% of women and 6.1% of men, and at the age of 55.0–64.9, the difference was already marked (35.9% vs. 15.0%). The occurrence of differences in excessive body weight depending on sex and age was confirmed with logistic regression analysis. Men were found to have 28% higher odds of becoming overweight than women. Regarding age as a differentiating criterion, it was shown that the OR (odds ratio) was 1.029. The prevalence of excessive body weight in Poland also depends on sociodemographic variables. Further analysis is required according to the type of work performed by the respondents and the physical activity undertaken, which may affect the body weight of the respondents, especially since low physical activity and a sedentary lifestyle resulting from professional activity are the risk factors for the development of obesity [29]. Simultaneously, being overweight and having obesity were found in nearly one in three rural residents, while the activity rate in rural and urban areas in Poland was 58.5% in 2022 [30]. The present study also showed that almost every second overweight person had secondary education, which might be considered in relation to the percentage of the professionally active population of Poland. In 2022, the group of people with secondary education included 55% of professionally active people, while the group with tertiary education included 81% of professionally active individuals [30]. At the same time, the results obtained in our study are consistent with the conceptual model of the “obesity transition” by Jaacks LM, et al. (2020). According to the concept, European countries are at “Stage 3” of the model that assumes that obesity is more common in people with lower socioeconomic status, e.g., they are more often in the group with a lower level of education compared to people with higher education [31]. Our study revealed a very high percentage of overweight people or people with obesity in the group declaring an average financial situation, which was also confirmed by the results by Stival Ch, et al. (2022). They demonstrated that the prevalence of obesity was associated with a lower level of education and socioeconomic status in European countries [32].

When discussing the issue of excessive body weight, it is worth noting the distribution of body fat. It is known that the accumulation of fat within the abdominal cavity is accompanied by an increased secretion of inflammatory cytokines by macrophages infiltrating the adipose tissue. It leads to the development of chronic low-grade inflammatory state in the body. Moreover, this type of obesity contributes to the reduction in tissue insulin sensitivity, the development of insulin resistance, and the risk of developing type 2 diabetes [33,34,35]. The obtained results indicated that every fifth adult resident of Poland had abdominal overweight, and nearly every third had abdominal obesity. The excessive accumulation of the adipose tissue in the visceral region is significantly more common in women. Based on the measurement of waist circumference, 22.6% of them had visceral overweight, and 44.3% had visceral obesity. Visceral overweight affected 18.8% of men and visceral obesity affected 18.0%.

It is worth paying attention to the incidence of abdominal obesity in women of menopausal age, which usually occurs between 45–55 years of age [36]. In this age range, over 38% of Polish women have been characterized by abdominal obesity, and the percentage of women with this disorder has been found to increase until the age of 75. It should be emphasized that excess visceral adipose tissue was found to have a stronger adverse effect on cardiovascular risk in women than in men [37]. Therefore, the high prevalence of the visceral distribution of the adipose tissue that we observed in women is a reason for concern.

The accumulation of the adipose tissue in the visceral area changes with the age of the respondents. It occurred in 32.5% of the respondents in the youngest group (19–34.9 years), and in the group of people aged 55–64.9 years, it was reported in 68.1% of the respondents. As regards the oldest age groups, waist circumference values were still too high in about 67% of people. In the years of 2013–2014, the prevalence of the excessive accumulation of the adipose tissue in the visceral region was higher than at present. At the beginning of the previous decade, abdominal overweight occurred in 21.7% of women and 27.2% of men, and abdominal obesity occurred in 48.7% of women and 32.2% of men. Moreover, waist circumference was too high in 91.8% of women (≥80 cm) and 77.2% of men (≥94 cm) at the age of 65–74.9. The excessive accumulation of the adipose tissue in the visceral region was reported even in the youngest age group (20–34.9 years), with 41.9% of women being affected. It is worth emphasizing that obesity in young women was associated with an increased risk of infertility caused by ovulation disorders [38], and the abdominal distribution of the adipose tissue was found to be linked with polycystic ovary syndrome [39,40,41]. Therefore, it may be assumed that obesity, especially abdominal, is at least partly responsible for such widespread fertility disorders in women nowadays. The problem of excessive body weight also affects a large percentage of young men. In our study, it occurred in 46.1% of men in the youngest age group and in 55% of those aged 35–45. Notably, it is also an important risk factor for infertility in the male population [42]. Thus, overweight/obesity in the partner should also be taken into account in the prevention and treatment of infertility in couples.

The results of the study suggest a reduction in the prevalence of general and abdominal obesity in the Polish population compared to research from 2013, which may indicate the effectiveness of population-scale preventive efforts. However, this observation needs to be confirmed through performing an additional analysis consisting in the standardization of the obtained results by age according to the demographic structure of the Polish population from 2013. Further analysis is required in terms of the diet and other lifestyle elements such as physical activity and length and quality of sleep, as these may help explain this beneficial change.

Our study also has some limitations. One of them was related to the change in the methods of collecting nutritional data—from the direct (in person) to the remote method (via telephone). During the pandemic period, the current anthropometric measurements were declared by the respondents (10% study group). However, the respondents were not asked about the exact measurement time. The rates of medical conditions were based on respondent declarations. The shift from in-person to telephone interviews might introduce inconsistencies in data quality. In addition, the respondents’ place of residence (city, village, etc.) and their financial situation require more detailed analyses.

## 5. Conclusions

Overweight cases and obesity, both general and abdominal, are still a major epidemiological problem in Poland.

The person with obesity/excess weight in the Polish population was most often a rural resident, aged 65.0–74.9, assessing their financial situation as average, being married/in a partnership, and not declaring the occurrence of cardiovascular diseases. The person with excess weight had asecondary education, and the person with obesity had primary/lower secondary/vocational education. The obtained results indicate that rural residents with lower education should be covered by educational activities adapted to their needs and possibilities.

Excessive body weight affected almost 42% of the examined women from the youngest age group, and abdominal obesity was found in 21% of women of this age. Further research and educational activities are necessary within the aspect of fertility, considering that obesity in young women, especially abdominal obesity, may be associated with an increased risk of infertility caused by ovulation disorders.

The results obtained in this study, when compared to previously published ones, suggest that the prevalence of being overweight and being obese has decreased over the last 10 years. However, this assumption requires further investigation.

## Figures and Tables

**Table 1:** Sociodemographic sample characteristics by sex in Poland (2017–2020) [*n* = 3735].

Variable	Women		Men		Total	χ^2^	*p*-Value ^a^
	*n*	%	*n*	%	*n*		
Age group							
19.0–34.9	241	22.4	245	24.9	486	14.830	0.011
35.0–44.9	204	19.0	231	23.5	435		
45.0–54.9	199	18.5	164	16.7	363		
55.0–64.9	131	12.2	107	10.9	238		
65.0–74.9	235	21.8	202	20.5	437		
≥75.0	66	6.1	36	3.7	102		
Place of residence							
Village	319	29.7	347	35.2	666	22.914	0.003
Towns to 10,000	89	8.3	76	7.7	165		
Towns 10–19 thousand	76	7.1	60	6.1	136		
Towns 20–49 thousand	130	12.1	112	11.4	242		
Towns 50–99 thousand	93	8.6	99	10.1	192		
Cities 100–199 thousand	93	8.6	68	6.9	161		
Cities 200–499 thousand	90	8.4	91	9.2	181		
Cities 500 thousand–1 million	95	8.8	89	9.0	184		
City > 1 million	91	8.5	43	4.4	134		
Education							
Primary/junior high school/vocational	342	31.8	424	43.1	766	43.337	<0.001
Secondary	518	48.1	450	45.7	968		
Tertiary	216	20.1	111	11.3	327		
Financial situation							
Good	205	19.1	292	29.6	497	32.631	<0.001
Average	763	70.9	619	62.8	1382		
Poor	108	10.0	74	7.5	182		
Marital status							
Unmarried	136	12.6	226	22.9	362	85.213	<0.001
Married/a cohabitation relationship	742	69.0	685	69.5	1427		
Divorced/separated	68	6.3	41	4.2	109		
Widow(er)	130	12.1	33	3.4	163		
Professional situation							
Retired/disability pensioner	379	35.2	279	28.3	658	11.259	0.001
Parental leave, unemployed, runs the house	86	8.0	27	2.7	113	27.366	<0.001
Casual employment	47	4.4	49	5.0	96	0.426	0.514
Permanent employment	551	51.2	620	62.9	1171	28.868	<0.001
Studies	27	2.5	22	2.2	49	0.169	0.681
Cardiovascular diseases ^b^							
No	927	86.2	882	89.5	1809	5.509	0.019
Yes	149	13.9	103	10.5	252		

^a^ chi-squared test, ^b^ hypertension, a history of stroke, a history of myocardial infarction, coronary heart disease, heart failure, atherosclerosis of lower extremity arteries, lipid disorders.

**Table 2:** BMI depending on the age and gender.

	Females (*n* = 1076)		Males (*n* = 985)		t	*p*-Value ^a^	Cohen’s *d* (95% CI)
	M (95% CI)	SD	M (95% CI)	SD			
BMI	26.56 (26.26; 26.86)	3.71	26.40 (26.18; 26.62)	5.17	0.804	0.421	0.034 (−0.048; 0.115)

BMI—body mass index; CI—confidence interval; M—mean; SD—standard deviation. ^a^ Student’s *t*-test.

**Table 3:** BMI depending on the age and gender.

BMI Categories	Females (*n* = 1076)		Males (*n* = 985)		z	*p*-Value ^a^	η^2^
	*n*	% (95% CI)	*n*	% (95% CI)			
<18.5	30	2.8 (1.8; 3.8)	1	0.1 (0.0; 0.3)	1.866	0.062	0.001
18.5–24.9	468	43.5 (40.5; 46.5)	393	39.9 (36.8; 43.0)			
25.0–29.9	363	33.7 (30.9; 36.5)	469	47.6 (44.5; 50.7)			
≥30.0	215	20.0 (17.6; 22.4)	122	12.4 (10.3; 14.5)			

CI—confidence interval; η^2^—eta squared for effect size ^a^ Mann–Whitney U test with tie correction.

**Table 4:** BMI categories by sex and age in Poland for 2017–2020.

Age Group	BMI Category	Women		Men		Total		z	*p*-Value ^a^	η^2^
		*n*	% (95% CI)	*n*	% (95% CI)	*n*	% (95% CI)			
19.0–34.9	<18.5	15	6.2 (3.2; 9.3)	0	0.0 (0.0; 1.5)	15	3.1 (1.4; 4.8)	−3.746	<0.001	0.014
	18.5–24.9	152	63.1 (57.0; 69.2)	132	53.9 (47.7; 60.1)	284	58.4 (54.0; 62.8)			
	25.0–29.9	53	22.0 (16.8; 27.2)	98	40.0 (33.9; 46.2)	151	31.1 (27.0; 35.2)			
	≥30.0	21	8.7 (5.1; 12.3)	15	6.1 (3.0; 9.2)	36	7.4 (5.2; 9.6)			
35.0–44.9	<18.5	7	3.4 (0.5; 6.3)	0	0.0 (0.0; 1.6)	7	1.6 (0.5; 3.7)	−1.270	0.204	0.003
	18.5–24.9	103	50.5 (43.9; 57.1)	104	45.0 (38.6; 51.4)	207	47.6 (42.9; 52.3)			
	25.0–29.9	67	32.8 (26.6; 39.0)	95	41.1 (34.8; 47.4)	162	37.2 (32.6; 41.8)			
	≥30.0	27	13.2 (8.5; 17.9)	32	13.9 (9.6; 18.2)	59	13.6 (10.4; 16.8			
45.0–54.9	<18.5	3	1.5 (0.0; 3.2)	0	0.0 (0.0; 2.3)	3	0.8 (0.1; 2.5)	−2.323	0.020	0.009
	18.5–24.9	92	46.2 (39.3; 53.1)	60	36.6 (29.5; 43.7)	152	41.9 (36.9; 46.9)			
	25.0–29.9	76	38.2 (31.5; 44.9)	83	50.6 (43.2; 58.0)	159	43.8 (38.8; 48.8)			
	≥30.0	28	14.1 (9.5; 18.7)	21	12.8 (7.8; 17.8)	49	13.5 (10.2; 16.8)			
55.0–64.9	<18.5	3	2.3 (0.0; 5.4)	0	0.0 (0.0; 3.4)	3	1.3 (0.0; 3.1)	2.014	0.044	0.010
	18.5–24.9	29	22.1 (15.0; 29.2)	32	29.9 (21.2; 38.6)	61	25.6 (20.1; 31.1)			
	25.0–29.9	52	39.7 (31.4; 48.0)	59	55.1 (45.7; 64.5)	111	46.6 (40.2; 53.0)			
	≥30.0	47	35.9 (27.7; 44.1)	16	15.0 (8.4; 21.6)	63	26.5 (21.0; 32.0)			
65.0–74.9	<18.5	2	0.9 (0.0; 2.7)	1	0.5 (0.0; 2.6)	3	0.7 (0.0; 1.8)	2.209	0.027	0.004
	18.5–24.9	72	30.6 (24.7; 36.5)	55	27.2 (21.2; 33.2)	127	29.1 (24.8; 33.4)			
	25.0–29.9	93	39.6 (33.6; 45.6)	112	55.5 (48.6; 62.4)	205	46.9 (42.3; 51.5)			
	≥30.0	68	28.9 (23.2; 34.6)	34	16.8 (11.7; 21.9)	102	23.3 (19.3; 27.3)			
	<22.0	18	7.7 (4.5; 10.9)	9	4.5 (1.7; 7.3)	27	6.2 (4.0; 8.4)	0.894	0.371	0.001
	22.0–27.0	95	40.4 (34.2; 46.6)	91	45.1 (38.3; 51.9)	186	42.6 (38.0; 47.2)			
	>27.0	122	51.9 (45.6; 58.2)	102	50.5 (43.6; 57.4)	224	51.3 (46.7; 55.9)			
≥75.0	<18.5	0	0.0 (0.0; 5.5)	0	0.0 (0.0; 9.7)	0	0.0 (0.0; 3.6)	3.258	0.001	0.014
	18.5–24.9	20	30.3 (19.0; 41.6)	10	27.8 (13.3; 42.3)	30	29.4 (20.4; 38.4)			
	25.0–29.9	22	33.3 (21.7; 44.9)	22	61.1 (45.5; 76.7)	44	43.1 (33.5; 52.7)			
	≥30.0	24	36.4 (24.6; 48.2)	4	11.1 (1.0; 21.2)	28	27.5 (18.6; 36.4)			
	<22.0	2	3.0 (0.0; 8.8)	1	2.8 (0.0; 8.2)	3	2.9 (0.0; 7.0)	1.712	0.087	0.004
	22.0–27.0	28	42.4 (30.4; 54.4)	19	52.8 (36.5; 69.1)	47	46.1 (36.6; 55.6)			
	>27.0	36	54.6 (42.2; 67.0)	16	44.4 (28.3; 60.5)	52	51.0 (41.3; 60.7)			
Total	<18.5	30	2.8 (1.8; 3.8)	1	0.1 (0.0; 0.4)	31	1.5 (1.0; 2.0)	1.866	0.062	0.001
	18.5–24.9	468	43.5 (40.5; 46.5)	393	39.9 (36.9; 42.9)	861	41.8 (39.7; 43.9)			
	25.0–29.9	363	33.7 (30.9; 36.5)	469	47.6 (44.6; 50.6)	832	40.4 (38.3; 42.5)			
	≥30.0	215	20.0 (17.6; 22.4)	122	12.4 (10.4; 14.4)	337	16.4 (14.8; 18.0)			
	<22.0	20	6.6 (4.0; 9.2)	10	4.2 (1.7; 6.7)	30	5.6 (3.7; 7.5)	1.780	0.075	0.002
	22.0–27.0	123	40.9 (35.3; 46.5)	110	46.2 (40.0; 52.4)	233	43.2 (39.0; 47.4)			
	>27.0	158	52.5 (46.8; 58.2)	118	49.6 (43.4; 55.8)	276	51.2 (47.0; 55.4)			

CI—confidence interval; η^2^—eta squared for the effect size; ^a^ Mann–Whitney U test with tie correction.

**Table 5:** The prevalence of abdominal overweight status and abdominal obesity by sex and age in Poland, 2017–2020.

Age Group	WC Category	Women		Men		Total		z	*p*-Value ^a^	η^2^
		*n*	% (95% CI)	*n*	% (95% CI)	*n*	% (95% CI)			
19.0–34.9	W < 80.0 M < 94.0	146	60.6 (54.4; 66.8)	183	74.7 (69.1; 80.3)	329	67.7 (63.5; 71.9)	6.194	<0.001	0.035
	W 80–87.9 M 94–101.9	44	18.3 (13.4; 23.2)	41	16.7 (12.1; 21.3)	85	17.5 (14.0; 21.0)			
	W ≥ 88.0 M ≥ 102.0	51	21.2 (16.0; 26.4)	21	8.6 (5.2; 12.0)	72	14.8 (11.5; 18.1)			
35.0–44.9	W < 80.0 M < 94.0	80	39.2 (32.6; 45.8)	156	67.5 (61.4; 73.6)	236	54.3 (49.5; 59.1)	8.130	<0.001	0.103
	W 80–87.9 M 94–101.9	39	19.1 (13.6; 24.6)	47	20.4 (15.1; 25.7)	86	19.8 (16.1; 23.5)			
	W ≥ 88.0 M ≥ 102.0	85	41.7 (35.0; 48.4)	28	12.1 (7.8; 16.4)	113	26.0 (22.0; 30.0)			
45.0–54.9	W < 80.0 M < 94.0	64	32.2 (25.8; 38.6)	93	56.7 (49.0; 64.4)	157	43.3 (38.2; 48.4)	5.712	<0.001	0.057
	W 80–87.9 M 94–101.9	59	29.7 (23.4; 36.0)	33	20.1 (13.9; 26.3)	92	25.3 (20.8; 29.8)			
	W ≥ 88.0 M ≥ 102.0	76	38.2 (31.5; 44.9)	38	23.2 (16.5; 29.9)	114	31.4 (26.8; 36.0)			
55.0–64.9	W < 80.0 M < 94.0	17	13.0 (7.2; 18.8)	59	55.1 (45.4; 64.8)	76	31.9 (25.9; 37.9)	7.500	<0.001	0.141
	W 80–87.9 M 94–101.9	31	23.7 (16.4; 31.0)	26	24.3 (16.1; 32.5)	57	24.0 (18.6; 29.4)			
	W ≥ 88.0 M ≥ 102.0	83	63.3 (55.0; 71.8)	22	20.6 (12.9; 28.3)	105	44.1 (37.9; 50.3)			
65.0–74.9	W < 80.0 M < 94.0	38	16.2 (11.5; 20.9)	110	54.5 (47.7; 61.3)	148	33.9 (29.5; 38.3)	15.331	<0.001	0.173
	W 80–87.9 M 94–101.9	54	23.0 (17.7; 28.3)	33	16.3 (11.3; 21.3)	87	19.9 (16.2; 23.6)			
	W ≥ 88.0 M ≥ 102.0	143	60.9 (54.8; 67.0)	59	29.2 (22.9; 35.5)	202	46.2 (41.5; 50.9)			
≥75.0	W < 80.0 M < 94.0	11	16.7 (8.2; 25.2)	22	61.1 (44.5; 77.7)	33	32.4 (23.2; 41.6)	8.966	<0.001	0.097
	W 80–87.9 M 94–101.9	16	24.2 (14.0; 34.4)	5	13.9 (1.8; 26.0)	21	20.6 (12.8; 28.4)			
	W ≥ 88.0 M ≥ 102.0	39	59.1 (47.0; 71.2)	9	25.0 (10.5; 39.5)	48	47.0 (37.3; 56.9)			
Total:	W < 80.0 M < 94.0	356	33.1 (30.3; 35.9)	623	63.2 (60.2; 66.4)	979	47.5 (45.4; 49.6)	23.071	<0.001	0.115
	W 80–87.9 M 94–101.9	243	22.6 (20.1; 25.1)	185	18.8 (16.4; 21.2)	428	20.8 (19.1; 22.5)			
	W ≥ 88.0 M ≥ 102.0	477	44.3 (41.3; 47.3)	177	18.0 (15.6; 20.4)	654	31.7 (29.8; 33.6)			

Abdominal overweight: Man WC 94–101.9 cm; Woman WC 80–87.9 cm. Abdominal obesity: Man WC ≥ 102; Woman WC ≥ 88 cm. CI—confidence interval, η^2^—eta squared for effect size. ^a^ Mann–Whitney U test with tie correction.

**Table 6:** Odds of becoming overweight/obesity (BMI ≥ 25.00 [19–64 yo] or BMI > 27.00 [65+ yo]) in Poland, 2017–2020.

	OR	−95% CI	+95% CI	Wald Stat.	*p*-Value
	0.263	0.209	0.332	127.175	<0.001
Sex (W vs. M)	1.288	1.126	1.473	13.620	<0.001
Age	1.029	1.025	1.033	228.830	<0.001
	OR ^a^	−95% CI	+95% CI	Wald stat.	*p*-value
	0.166	0.077	0.358	20.988	0.000
Sex (W vs. M)	1.278	1.101	1.484	10.341	0.001
Age	1.020	1.011	1.028	21.407	0.000

CI—confidence interval; OR—odds ratio; ^a^ adjusted odds ratio: region, place of residence, education, economic situation, marital status of respondents, professional situation, and the presence of cardiovascular disease.

**Table 7:** Profile of an overweight woman/man and a woman/man with obesity.

Profile of an Overweight Woman/Man: BMI 25.00–29.90 [*n* = 863, 41.9% of the Population]							
Variable	Women		Men		Total	χ^2^	*p*-Value ^a^
	*n*	%	*n*	%	*n*		
Place of residence							
Village	99	27.1	176	35.4	275	37.428	<0.001
Towns to 10,000	20	5.5	25	5.0	45		
Towns 10–19 thousand	29	7.9	32	6.4	61		
Towns 20–49 thousand	48	13.1	50	10.1	98		
Towns 50–99 thousand	25	6.8	57	11.5	82		
Cities 100–199 thousand	42	11.5	22	4.4	64		
Cities 200–499 thousand	29	7.9	48	9.7	77		
Cities 500 thousand–1 million	33	9.0	60	12.1	93		
City > 1 million	41	11.2	27	5.4	68		
Age group							
19.0–34.9	53	14.5	107	21.5	160	9.082	0.106
35.0–44.9	67	18.3	100	20.1	167		
45.0–54.9	77	21.0	85	17.1	162		
55.0–64.9	53	14.5	66	13.3	119		
65.0–74.9	94	25.7	115	23.1	209		
≥75.0	22	6.0	24	4.8	46		
Education							
Primary/junior high school/vocational	126	34.4	213	42.9	339	20.593	<0.001
Secondary	164	44.8	234	47.1	398		
Tertiary	76	20.8	50	10.1	126		
Financial situation							
Good	60	16.4	153	30.8	213	26.232	<0.001
Average	263	71.9	310	62.4	573		
Poor	43	11.8	34	6.8	77		
Marital status							
Unmarried	32	8.7	89	17.9	121	32.003	<0.001
Married/a cohabitation relationship	263	71.9	365	73.4	628		
Divorced/separated	25	6.8	19	3.8	44		
Widow(er)	46	12.6	24	4.8	70		
Professional situation							
Retired/disability pensioner	147	40.2	165	33.2	312	4.429	0.035
Parental leave, unemployed, runs the house	22	6.0	10	2.0	32	9.440	0.002
Casual employment	14	3.8	13	2.6	27	1.017	0.313
Permanent employment	183	50.0	308	62.0	491	12.318	<0.001
Studies	4	1.1	5	1.0	9	0.015	0.901
Cardiovascular diseases ^b^							
No	308	84.2	436	87.7	744	2.264	0.132
Yes	58	15.9	61	12.3	119		
**Profile of a woman/man with obesity bmi ≥30 [*n* = 350, 17.0% of the population]**							
**Variable**	**Women**		**Men**		**Total**	**χ^2^**	***p*-Value** **^a^**
	***n***	**%**	***n***	**%**	***n***		
Place of residence							
Village	62	28.31	43	32.8	105	17.799	0.023
Towns to 10,000	16	7.31	11	8.4	27		
Towns 10–19 thousand	13	5.94	3	2.3	16		
Towns 20–49 thousand	17	7.76	12	9.2	29		
Towns 50–99 thousand	23	10.50	13	9.9	36		
Cities 100–199 thousand	20	9.13	9	6.9	29		
Cities 200–499 thousand	16	7.31	17	13.0	33		
Cities 500 thousand–1 million	27	12.33	21	16.0	48		
City > 1 million	25	11.42	2	1.5	27		
Age group							
19.0–34.9	21	9.59	17	13.0	38	20.046	0.001
35.0–44.9	27	12.33	34	26.0	61		
45.0–54.9	31	14.16	23	17.6	54		
55.0–64.9	48	21.92	18	13.7	66		
65.0–74.9	68	31.05	35	26.7	103		
≥75.0	24	10.96	4	3.1	28		
Education							
Primary/junior high school/vocational	104	47.49	63	48.1	167	2.112	0.348
Secondary	92	42.01	48	36.6	140		
Tertiary	23	10.50	20	15.3	43		
Financial situation							
Good	28	12.79	42	32.1	70	19.447	<0.001
Average	164	74.89	79	60.3	243		
Poor	27	12.33	10	7.6	37		
Marital status							
Unmarried	12	5.48	22	16.8	34	27.385	<0.001
Married/a cohabitation relationship	144	65.75	96	73.3	240		
Divorced/separated	16	7.31	7	5.3	23		
Widow(er)	47	21.46	6	4.6	53		
Professional situation							
Retired/disability pensioner	122	55.71	43	32.8	165	17.225	<0.001
Parental leave, unemployed, runs the house	19	8.68	5	3.8	24	3.030	0.082
Casual employment	7	3.20	4	3.1	11	0.005	0.941
Permanent employment	72	32.88	82	62.6	154	29.381	<0.001
Studies	1	0.46	0	0.0	1	0.600	0.439
Cardiovascular diseases ^b^							
No	163	74.43	100	76.3	263	0.160	0.690
Yes	56	25.57	31	23.7	87		

^a^ chi-squared test. ^b^ hypertension, a history of stroke, a history of myocardial infarction, coronary heart disease, heart failure, atherosclerosis of lower extremity arteries, lipid disorders.

## Data Availability

Dataset available on request from the authors.

## Supplementary Materials

The following supporting information can be downloaded at: https://www.mdpi.com/article/10.3390/nu16234248/s1, File S1: Detailed Diagnostics of Logistic Regression Models.
