# Supplementary material for: Prevalence of Overweight, Obesity, and Abdominal Obesity in Polish Adults: Sociodemographic Analysis from the 2016–2020 National Health Program
Source: Nutrients. 2024 Dec 9;16(23):4248. doi: 10.3390/nu16234248 (PMC11644767; doi:10.3390/nu16234248)
Supplement: Supplementary file 1 [file nutrients-16-04248-s001.zip › nutrients-3350655-supplementary.pdf]

Supplementary Materials:

File S1. Detailed Diagnostics of Logistic Regression Models

Model Fit Measures

		Overall Model Test		
AIC	R^2_N	χ^2	df	p
4602	0.0987	274	2	<0.001

Omnibus Likelihood Ratio Tests

Predictor	χ^2	df	p
Age	272.38	1	<0.001
Sex	8.69	1	0.003

Model Coefficients - Overweight / obesity

		95% Confidence Interval			
Predictor	Odds ratio	Lower	Upper	Wald stat.	p
Intercept	0.263	0.209	0.332	127.175	<0.001
Sex:					
M – W	1.288	1.126	1.473	13.620	<0.001
Age	1.029	1.025	1.033	228.830	<0.001

Note. Estimates represent the log odds o[illegible]"Overweight / obesity = 1" vs. "Overweight / obesity = 0"

Collinearity Statistics

	VIF	Tolerance
Age	1.01	0.987
Sex	1.01	0.987

Note. Variance Inflation Factor

Model Fit Measures

		Overall Model Test		
AIC	R^2_N	χ^2	df	p
4419	0.179	515	31	<0.001

Omnibus Likelihood Ratio Tests

Predictor	χ^2	df	p
Age	28.894	1	< .001
Sex	6.950	1	0.008
Region	138.542	15	< .001
Education	6.499	2	0.039
Place of residence	0.804	1	0.370
Economic situation	2.329	2	0.312
Marital status of respondents	17.626	3	< .001
Pension	4.423	1	0.035
Vacation	5.047	1	0.025
Part-time work	1.086	1	0.297
Employment	6.437	1	0.011
Studying	0.001	1	0.973
Presence of cardiovascular disease	45.448	1	< .001

Model Coefficients - Overweight / obesity

		95% Confidence Interval			
Predictor	Odds ratio*	Lower	Upper	Wald stat.	p
Intercept	0.166	0.077	0.358	20.988	0.000
Sex:					
M – W	1.278	1.101	1.484	10.341	0.001
Age	1.020	1.011	1.028	21.407	0.000

Note. Estimates represent the log odds of "Overweight / obesity = 1" vs. "Overweight / obesity = 0"
* adjusted Odds Ratio: region, place of residence, education, economic situation, marital status of respondents, professional situation and the presence of cardiovascular disease

Collinearity Statistics

	VIF	Tolerance
Age	2.19	0.457
Sex	1.09	0.917
Region	1.02	0.985
Education	1.08	0.927
Place of residence	1.04	0.965
Economic situation	1.08	0.925
Marital status of respondents	1.13	0.886
Pension	4.2	0.238
Vacation	1.94	0.516
Part-time work	1.49	0.672
Employment	4.06	0.247
Studying	1.41	0.71
Presence of cardiovascular disease	1.08	0.925

Note. Variance Inflation Factor
